# Acceptability of Using a Robotic Nursing Assistant in Health Care Environments: Experimental Pilot Study

**DOI:** 10.2196/17509

**Published:** 2020-11-12

**Authors:** Mohammad Nasser Saadatzi, M Cynthia Logsdon, Shamsudeen Abubakar, Sumit Das, Penelope Jankoski, Heather Mitchell, Diane Chlebowy, Dan O Popa

**Affiliations:** 1 Electrical and Computer Engineering Department University of Louisville Louisville, KY United States; 2 School of Nursing University of Louisville Louisville, KY United States; 3 Biomedical Engineering Department Case Western Reserve University Cleveland, OH United States

**Keywords:** robot-assisted healthcare, robotic nurse assistant, technology acceptance model, user acceptability, patient walking

## Abstract

**Background:**

According to the US Bureau of Labor Statistics, nurses will be the largest labor pool in the United States by 2022, and more than 1.1 million nursing positions have to be filled by then in order to avoid a nursing shortage. In addition, the incidence rate of musculoskeletal disorders in nurses is above average in comparison with other occupations. Robot-assisted health care has the potential to alleviate the nursing shortage by automating mundane and routine nursing tasks. Furthermore, robots in health care environments may assist with safe patient mobility and handling and may thereby reduce the likelihood of musculoskeletal disorders.

**Objective:**

This pilot study investigates the perceived ease of use and perceived usefulness (acceptability) of a customized service robot as determined by nursing students (as proxies for nursing staff in health care environments). This service robot, referred to as the Adaptive Robotic Nurse Assistant (ARNA), was developed to enhance the productivity of nurses through cooperation during physical tasks (eg, patient walking, item fetching, object delivery) as well as nonphysical tasks (eg, patient observation and feedback). This pilot study evaluated the acceptability of ARNA to provide ambulatory assistance to patients.

**Methods:**

We conducted a trial with 24 participants to collect data and address the following research question: Is the use of ARNA as an ambulatory assistive device for patients acceptable to nurses? The experiments were conducted in a simulated hospital environment. Nursing students (as proxies for nursing staff) were grouped in dyads, with one participant serving as a nurse and the other acting as a patient. Two questionnaires were developed and administrated to the participants based on the Technology Acceptance Model with respect to the two subscales of perceived usefulness and perceived ease of use metrics. In order to evaluate the internal consistency/reliability of the questionnaires, we calculated Cronbach alpha coefficients. Furthermore, statistical analyses were conducted to evaluate the relation of each variable in the questionnaires with the overall perceived usefulness and perceived ease of use metrics.

**Results:**

Both Cronbach alpha values were acceptably high (.93 and .82 for perceived usefulness and perceived ease of use questionnaires, respectively), indicating high internal consistency of the questionnaires. The correlation between the variables and the overall perceived usefulness and perceived ease of use metrics was moderate. The average perceived usefulness and perceived ease of use metrics among the participants were 4.13 and 5.42, respectively, out of possible score of 7, indicating a higher-than-average acceptability of this service robot.

**Conclusions:**

The results served to identify factors that could affect nurses’ acceptance of ARNA and aspects needing improvement (eg, flexibility, ease of operation, and autonomy level).

## Introduction

### Background

According to the American Association of Colleges of Nursing, there is a shortage of registered nurses (RNs) in the United States, which is expected to escalate due to the increase in health care demands and needs of baby boomers as they age [1]. Robot-assisted health care has the potential to mitigate this shortage by automating mundane and routine nursing tasks, thereby enhancing the productivity and efficiency of nurses [2]. RNs are the largest group of staff in US health care systems, and they experience an above-average incidence rate of physical injuries and musculoskeletal disorders [3]. In 2016, the incidence rate of musculoskeletal disorders in RNs was 46.0 cases in every 10,000 workers, which was substantially higher than the average incidence rate of other occupations at 29.4 cases in every 10,000 workers [4]. Overexertion and bodily reaction accounted for 49.7% of the cases among RNs working in hospitals, 35.0% among RNs in ambulatory health care services, and 34.5% among RNs in nursing and residential care facilities [4]. The American Nurses Association initiated a national campaign in 2003 to decrease the number of musculoskeletal injuries in nurses [5] and subsequently published a guideline to advise nurses on how to avoid injuries while handling patients, which included the use of available technology [6]. However, the incidence of musculoskeletal injuries in nurses remains high.

Robots may assist with safe patient handling and mobility in health care environments. In recent years, robots have been used in hospitals to assist with surgical procedures, deliver medications, monitor patients, and assist with daily hygiene [7]. However, nurses’ acceptance of robotic nursing assistants is essential and warrants comprehensive assessment in order to ensure the adoption of this technology and its large-scale implementation in health care environments. While a few surveys of professionals have demonstrated positive feedback related to the use of robots in health care [8,9], feasibility data is needed in order to assess how robots can assist with safe patient handling and mobility, thereby preventing musculoskeletal injuries among nurses.

**Figure 1:**
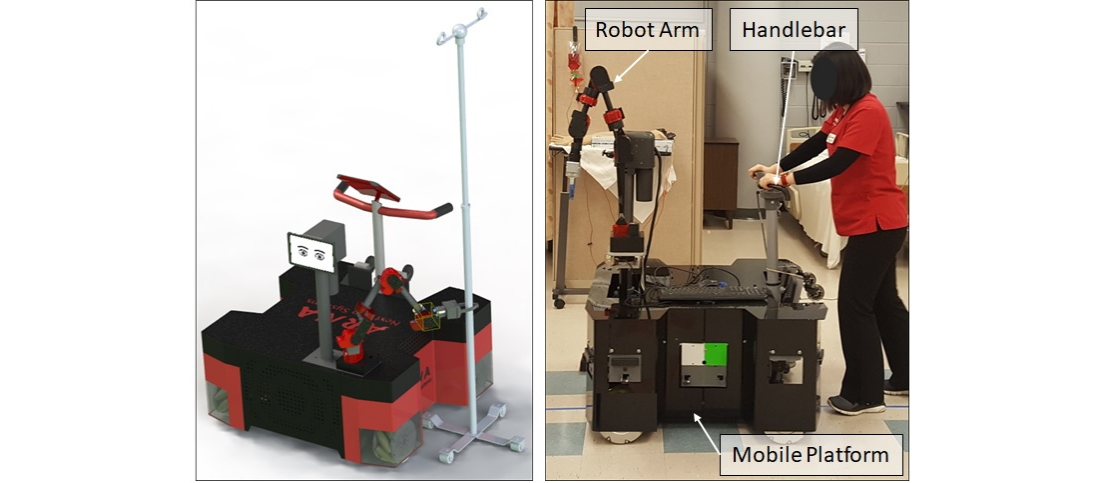
Adaptive robotic nursing assistant including an omnidirectional mobile platform, a 6-DoF robotic arm, and an instrumented handlebar.

In order to off-load some of the physically demanding tasks assigned to nursing staff and prevent physical injuries, we have built a robotic nursing assistant, referred to as Adaptive Robotic Nursing Assistant (ARNA). ARNA is a custom-built service robot, which is capable of autonomous navigation in hospital environments and performing tasks as a nursing assistant. ARNA is an omnidirectional mobile robot constructed in-house and augmented with a 6-DoF (degrees of freedom) robotic arm (Figure 1). ARNA is equipped with an instrumented handlebar that can detect a patient’s navigational intent when used as a patient walker. ARNA has been designed to enhance the productivity of nursing staff through cooperation during physical tasks and nonphysical tasks (eg, patient walking, item fetching, patient observation, and collecting patient feedback).

### Research Question

This study evaluated the acceptability of ARNA as determined by nursing students in scenarios where ARNA was used as a robotic patient walker. The experiments were conducted in a simulated hospital environment. Nursing students (as proxies for nursing staff) were grouped in dyads, with one participant serving as a nurse and the other acting as a patient. We conducted a phase 1 trial (ie, pilot study) to collect quantitative data and address the following research question: Is the use of a service robot as an ambulatory assistive device for patients acceptable to nurses? In this context, user acceptability was measured using the two subscales of the Technology Acceptance Model (TAM) [10,11], that is, perceived usefulness and perceived ease of use.

## Methods

### Participants

All nursing students (n=24) were enrolled in coursework in undergraduate and master’s entry accelerated second-degree nursing programs that included patient care in hospitals. Recruitment occurred through in-class invitations by nursing faculty. Subsequently, interested students notified the faculty to volunteer for the study. As compensation for participating in the experiments, students received course credit for clinical/research hours for an undergraduate research course or capstone clinical course.

Student volunteers were required to have the physical ability to adequately perform the tasks administered in the study. It should be noted that disabilities were used as exclusion criteria only if the disability substantially interfered with the performance of the tasks (eg, noncorrectable vision or hearing problems). Approval to conduct the study was obtained from the university’s institutional review board. All volunteers gave informed consent.

### Robot Description

ARNA is an omnidirectional mobile robot constructed in-house and augmented with a 6-DoF robotic arm (Figure 1). ARNA is also equipped with a handlebar instrumented with a force-torque sensor, which enables the robot to detect the user’s navigational intent and adjust the amount of physical assistance while walking patients or pushing heavy items (eg, hospital beds). ARNA includes multiple user interfaces (eg, tablet interface, gamepad-like remote control), navigation sensors (eg, cameras, Kinect, ultrasonic and infrared sensors), and a powerful computing and real-time control unit. Although ARNA has several functionalities and services, the current study focused on the user acceptability of ARNA when used as a robotic patient walker. Acceptability data of other functions of ARNA will be investigated in a subsequent study.

The prevention of patient falls is a measure of quality care in hospitals and a priority in the daily work of nurses. When ARNA is utilized as a patient walker, the user interaction efforts (forces and torques applied to the handlebar by patient) are sensed by the force-torque sensor mounted underneath the ARNA’s handlebar and used to extract the user’s navigational intent. The user intent is then communicated to the robot’s control unit, which, in turn, propels the robot in the intended direction, thereby inducing the feeling in users that they are interacting with a light-weight walker. Apart from providing ambulation assistance and stability support, ARNA utilizes its robotic arm to carry along intravenous (IV) lines and other tethering medical equipment (eg, oxygen cylinder). During such assisted walking, ARNA’s navigation sensors enhance the user’s safety by avoiding collisions of the robot with the carried IV pole and with objects and humans in the environment. From a technical standpoint, a high level of intelligence and autonomy is required to collaborate with a nurse and perform these tasks. For this purpose, we have equipped ARNA with a variety of sensors including cameras, Kinect, laser scanner, bump sensors, infrared sensors, and ultrasonic sensors.

### Experimental Setting

The experiments were conducted at a simulation suite located at School of Nursing, University of Louisville.

### Trial Design and Procedure

Before participants arrived at the experiment location, the setting and robot were prepared by the project personnel. Only 2 nursing students and the project personnel were in the simulation lab at any given time. To facilitate task comprehension, participants received thorough verbal instructions and a task demonstration regarding the experiment procedure.

Participants worked in dyads, with one participant serving as a nurse and the other acting as a patient requiring walking assistance. A rectangular path was marked on the floor for the participants to follow while receiving gait assistance from ARNA. The general task of “patient” was to hold onto the robot handlebar and walk with the robot while following the marked path on the floor, and the task of “nurse” was to walk alongside the patient and robot in a supervisory role holding an emergency stop switch. While walking, ARNA carried along an IV pole using its arm, maintaining a safe distance between the pole and objects in the environment, the patient, and itself. The nurse was instructed to activate the emergency stop switch, which would bring the robot to a complete hold, should the robot come into close vicinity of other objects or any unforeseen dangerous circumstances. Before beginning of the trial, the patient reclined in a hospital bed, and the nurse stood by him/her, both waiting for the trial initiation. Pursuant to the initiation signal from the experimenter, the patient received assistance from the nurse to climb down the bed and hold onto the ARNA’s handlebar. When the patient verbally confirmed firm grasp of the handlebar and stable posture, the nurse deactivated ARNA’s emergency stop, allowing the robot to operate. Next, the patient followed the rectangular path marked on the floor while receiving support from the robot until he/she arrived back at the bed, at which point the nurse reactivated the emergency stop and helped the patient lie back in the bed. Each experiment set consisted of 9 trial runs per participant. Original dyads were retained for all trial runs.

Following the ninth trial, the nursing students were given a survey (perceived usefulness and perceived ease of use subscales of the TAM) about their experience interacting with the robot and assisting the patient. Surveys were administered in the form of a questionnaire and as Likert scales (discussed in the following subsection). Subsequently, the participants’ roles were switched, thus providing the roles of nurse and patient to both participants, and another 9-trial set of experiment runs repeated for a total of 216 trial runs. The entire experiment lasted 6 days (2 dyads per day). Participant groupings and nurse/patient role orders were randomized to prevent any inherent biases.

### Theoretical Framework

The TAM was originally developed by Davis in 1989 to predict and explain a user’s acceptance of information technology (IT) [10]. The original TAM was composed of two fundamental constructs hypothesized to be determinants of user acceptance: perceived usefulness and perceived ease of use [10]. The Theory of Reasoned Action provided the theoretical framework for the TAM to explain the relationships between perceived usefulness and perceived ease of use as well as users’ attitudes, intentions, and IT use behavior [11]. After the application of the TAM in IT workplace studies, researchers suggested that the TAM include external variables related to social change, human processes, and boundary-related conditions [12,13]. In 2003, the original TAM was modified. The new TAM2 removed the concept of a*ttitudes* and broke down the concept of *external variables* into cognitive instrumental processes (perceived ease of use , job relevance, output quality) and social influence processes (image, subjective norm, voluntariness) [14]. The TAM3, an integrated model combining the TAM2 and the model of the determinants of perceived ease of use, was later developed in 2008 [15]. The TAM3 was composed of four constructs: perceived ease of use, perceived usefulness, use behavior, and behavior intention [15,16], although it has not found widespread use [17]. When reviewing the iterations of the TAM, the investigators decided that the original TAM best captured the concept of acceptability in this study.

This study used two subscales of the TAM (ie, perceived usefulness and perceived ease of use), originally proposed by Davis in 1989 [10], to evaluate acceptability and adoption likelihood of the ARNA robot by nursing students. The TAM has effectively predicted nurses’ acceptance of other health care technology [18]. For investigation of technology adoption in health care, the TAM is considered the gold standard and is thought to provide a strong validity and reliability measure [19]. As mentioned previously, the TAM evaluates user acceptability of a given technology via two subscales: perceived usefulness and perceived ease of use. Perceived usefulness is the degree to which one thinks using a specific system facilitates their job. Perceived ease of use , on the other hand, is the degree to which one thinks usage of that system is effort-free [10]. User acceptability of a variety of technologies (eg, service robots [18], e-learning systems [18,20,21], and assistive social robots [22]) have been evaluated by employing the TAM. To the best of our knowledge, this study is the first to gauge acceptance of service robots by nurses via perceived usefulness and perceived ease of use subscales of the TAM. The questionnaires are tabulated in Table 1 and 2. Each question was evaluated on a 7-point Likert scale, ranging from 1 (“disagree”) to 7 (“agree”).

**Table 1:** Results of the perceived usefulness subscale questionnaire.

Item	Mean (SD)	Correlation with overall usefulness	*P* value
1. Using ARNA robot improves my job performance.	3.75 (1.67)	0.45	.03
2. ARNA^a^ robot enables me to accomplish tasks more quickly.	4.08 (2.04)	0.60	.002
3. Using ARNA robot enhances my effectiveness on the job.	4.08 (1.9)	0.62	.001
4. Using ARNA makes it easier to do my job.	4.04 (1.97)	0.95	<.001
5. Using ARNA increases productivity.	4.5 (1.74)	0.69	<.001
6. Overall, I find ARNA useful.	4.13 (1.92)	1	<.001

^a^ARNA: Adaptive Robotic Nursing Assistant.

**Table 2:** Results of the perceived ease of use subscale questionnaire.

Item	Mean (SD)	Correlation with overall ease of use	*P* value
7. I do not become confused when I use ARNA.^a^	4.83 (1.43)	0.39	.054
8. I do not get frustrated when interacting with ARNA.	4.58 (1.58)	0.65	<.001
9. The system is flexible to work with.	3.54 (1.79)	0.51	.01
10. My interaction with ARNA is easy to understand.	5.83 (1.37)	0.58	.002
11. It is easy to remember how to perform tasks while using ARNA.	6.00 (1.35)	0.36	.08
12. Overall, I find ARNA easy to use.	5.42 (1.52)	1	<.001

^a^ARNA: Adaptive Robotic Nursing Assistant.

### Data Analysis

In order to examine the internal consistency of perceived usefulness and perceived ease of use subscales of the TAM in this context, we calculated Cronbach alpha values [23]. Cronbach alpha is a measure of scale reliability and examines whether a group of observations are closely related. A Cronbach alpha value greater than .7 is considered acceptable, a value below .2 is unreliable, and negative values indicate a completely random set [23]. In addition, we performed a correlation analysis to determine the relationships between questionnaire items and the overall perceived usefulness and perceived ease of use by computing the Spearman rho correlation coefficient and *P* values [24].

## Results

We calculated a Cronbach alpha of .93 for the perceived usefulness dataset and .82 for the perceived ease of use dataset.

The mean and SD for each item in the questionnaires, among the participants, are shown in Table 1 and 2. The mean values for overall perceived usefulness and perceived ease of use were 4.13/7 and 5.42/7, respectively, indicating moderate-to-high acceptability. According to Tables 1 and 2, the correlation coefficients between the individual questions and the overall perceived usefulness and perceived ease of use were moderate. Furthermore, the overall perceived usefulness and perceived ease of use were moderately correlated (Spearman rho=0.44; *P* value=.03). Additionally, weak-to-strong multicollinearities were detected between the perceived usefulness and perceived ease of use variables. In particular, variables 1, 2, 3, 7, 8, and 9 exhibited weak multicollinearity (condition index<10), variables 4 and 6 had a moderate collinearity (condition index=43), and variables 10 and 11 demonstrated a fairly strong collinearity (condition index=70).

## Discussion

### Principal Findings

This study evaluated the usefulness and ease of use of ARNA as perceived by nursing students in order to assess its acceptability and the influencing factors, as well as to identify areas needing improvement for future development of ARNA. The calculated Cronbach alpha (0.93 for perceived usefulness dataset and 0.82 for perceived ease of use dataset) demonstrates acceptable internal consistency of the questionnaires. The statistical findings of the perceived usefulness and perceived ease of use subscales indicate a moderate-to-high acceptance of ARNA by the nursing students. The general opinion of the participants about ARNA was positive, as the mean value for overall usefulness and ease of use were above average (ie, greater than 4/7). Furthermore, the correlation coefficients between the individual questions and the overall perceived usefulness and perceived ease of use were moderately acceptable, suggesting the usefulness of the questions in examining various aspects of perceived usefulness and perceived ease of use metrics.

In general, this study enhances our understanding of various aspects of ARNA in terms of its perceived usefulness and perceived ease of use and points out key aspects that require improvement in order to facilitate ARNA’s acceptability and adoption likelihood. For example, it is important to improve ARNA’s flexibility as suggested by its moderate mean value of users’ responses to question 9. Perhaps by providing more user interfaces to accommodate various interaction styles, nurses’ perceptions of flexibility could be enhanced. The participants’ responses to the other perceived ease of use questions, however, were fairly high, suggesting that the robot is easy to operate. This may be attributable to the fact that the robot autonomously regulates the gait speed, provides stability support and collision avoidance, and only requires nurses to supervise the gait assistance and respond in case of emergency situations.

The mean participant response to variable 1 was fairly low, and the mean responses to variables 2-5 were moderate, indicating that participants did not completely agree that use of ARNA robot improves their job performance or saves them substantial time. This is, however, not surprising, as in its current capacity, ARNA still requires a nurse to accompany the robot and the patient during the walking assistance, which is as time consuming as using a nonrobotic walker, although the robot provides stability support and collision avoidance. Endowing the robot with a fully autonomous capability to provide walking assistance to patients without direct nurse supervision may enhance users’ perception regarding job performance improvement. Completely outsourcing the walking assistance to a fully autonomous robot may render a nurse more efficient as he/she would only need to oversee the gait assistance and would therefore be able to focus on other responsibilities.

Simulation is a growing trend in health care education, and proxies are frequently needed to collect data in clinical settings for children or incapacitated patients. Preliminary acceptability data was needed before taking ARNA into a hospital or other clinical setting and testing it with busy health care providers and vulnerable patients. This simulated health care study using nursing students is viewed as a step forward in the evolution of ARNA as a tool for safe and quality patient care and as protection against musculoskeletal disorders among nurses.

### Limitations

The outcomes of this study should be deemed preliminary for a number of reasons. First, we evaluated usefulness and ease of use of ARNA with a limited number of nursing students in a simulated hospital environment, and, hence, the results may not extend to the whole population of nurses and health care providers in real health care environments. Second, due to our experimental design, each participant served as both patient and nurse, which may have influenced the participants’ perception of the acceptability of ARNA. This experimental design may also have affected the participants’ decisions during the study, thereby affecting the study results.

In addition, in this study, we were not able to collect and evaluate actual user behavior of the ARNA robot over a long time period due to the study’s preliminary nature, and, hence, perceived usefulness and perceived ease of use were used as proxies to predict adoption likelihood and usage of ARNA among nurses.

### Conclusion

The rate of physical injury and musculoskeletal disorders in nurses is higher than average as compared to other occupations, which is typically due to patient handling and mobility. Robots bear a great potential to off-load some of the physically demanding tasks of nursing staff and health care providers, and hence prevent physical injuries. However, nurses’ acceptance of robotic nursing assistants is essential and warrants comprehensive assessment in order to ensure adoption of this technology and its large-scale implementation in health care environments.

This study evaluated the acceptability of our custom-made robotic nursing assistant, ARNA, with respect to the two subscales of the TAM, perceived usefulness and perceived ease of use , during a patient walking scenario. User surveys were administered to 24 nursing students who served as the study participants. We conducted the experiments in a simulated hospital environment, where 2 participants were paired together, one playing the role of a patient and the other acting as a nurse providing ambulatory assistance to the patient using a service robot that functioned as a powered walker. After the trials, the participants serving as nurses filled out questionnaires about their opinions regarding usefulness and ease of use of the ARNA robot. This study, to the best of our knowledge, is the first to examine the user acceptability of robotic nursing assistants, serving as a stepping-stone for further discussions about the sociotechnical and commercial landscape surrounding this technology.

## Abbreviations

ARNA: Adaptive robotic nursing assistant
DoF: Degrees of freedom
RN: Registered nurses
TAM: Technology acceptance model
